# The effect of a self-constructed material on children’s physical activity during recess

**DOI:** 10.1590/S1518-8787.2017051006659

**Published:** 2017-06-20

**Authors:** Antonio Méndez-Giménez, José-Antonio Cecchini, Javier Fernández-Río

**Affiliations:** I Departamento de Ciencias de la Educación. Facultad de Formación del Profesorado y Educación. Universidad de Oviedo. Oviedo, España

**Keywords:** Child, Motor Activity, Play and Playthings, Participative Planning, Recreation, Health Behavior, Health Promotion

## Abstract

**OBJECTIVE:**

To analyze whether an intervention supported by free play with a self-constructed material increases the level of physical activity of students during recess.

**METHODS:**

The participants were 166 children of third to sixth grade, between nine and 12 years old (average = 10.64; SS = 1.13). An experimental project was conducted with pre-test and post-test measurement, and a control group. Experimental group participants built cardboard paddles (third and fourth) and flying rings (fifth and sixth), a material they used freely for one week during recess. ActiGraph-GT3X accelerometers were used to measure physical activity. An ANOVA of repeated measures was used to find differences between groups and genders.

**RESULTS:**

Significant intervention effects were found in the analyzed variables: sedentary activity (F = 38.19; p < 0.01), light (F = 76.56; p < 0.01), moderate (F = 27.44; p < 0.01), vigorous (F = 61.55; p < 0.01), and moderate and vigorous (F = 68.76; p < 0.01). Significant gender differences were shown (time × group × gender) for moderate (F = 6.58; p < 0.05) and vigorous (F = 5.51; p < 0.05) activity.

**CONCLUSIONS:**

The self-constructed material is effective to increase the physical activity levels of children during recess; it decreases sedentary activity and light physical activity and increases the time devoted to moderate physical activity and vigorous physical activity, both in boys and in girls. The boys had an increase in vigorous physical activity and the girls in moderate physical activity. Due to its low cost, this strategy is recommended for administrators and teachers to increase the physical activity of children during recess.

## RESUMEN

**OBJETIVO:**

Analizar si una intervención basada en el juego libre con material autoconstruido aumenta el nivel de actividad física de los escolares durante el recreo.

**MÉTODOS:**

Participaron 166 niños de tercero a sexto de educación primaria, de entre nueve y 12 años de edad (media = 10,64; DE = 1,13). Se realizó un diseño experimental con medidas pretest y postest, y un grupo control. Los participantes del grupo experimental construyeron palas de cartón (tercero y cuarto) y aros voladores (quinto y sexto), material que usaron libremente durante una semana en los recreos. Se utilizaron acelerómetros ActiGraph-GT3X para medir la actividad física. Se usó un ANOVA de medidas repetidas para investigar las diferencias entre grupos y sexo.

**RESULTADOS:**

Se encontraron efectos de intervención significativos en las variables analizadas: actividad sedentaria (F = 38,19; p < 0,01), ligera (F = 76,56; p < 0,01), moderada (F = 27,44; p < 0,01), vigorosa (F = 61,55; p < 0,01), y moderada y vigorosa (F = 68,76; p < 0,01). Emergieron diferencias significativas de sexo (tiempo x grupo x sexo) para la actividad moderada (F = 6,58; p < 0,05) y vigorosa (F = 5,51; p < 0,05).

**CONCLUSIONES:**

El material autoconstruido es eficaz para aumentar los niveles de actividad física de los niños en el recreo; disminuye la actividad sedentaria y la actividad física ligera, y aumenta el tiempo dedicado a la actividad física moderada y actividad física vigorosa, tanto en varones como en mujeres. Los varones aumentaron más la actividad física vigorosa y las mujeres, la actividad física moderada. Por su bajo coste, se recomienda esta estrategia a gestores y profesores para incrementar la actividad física de los niños durante el recreo.

## INTRODUCTION

Numerous studies have evaluated the benefits of regular physical activity (PA) for youth and child population health^11^. The World Health Organization (WHO, 2010)^27^ recommends 60 minutes or more of moderate or vigorous-intensity daily PA, mostly aerobics, for child and adolescent population between five and 17 years old. At the same time, the WHO suggests the inclusion of muscular strengthening exercises at least three days a week. However, a sizeable proportion of children and adolescents does not perform PA at adequate levels to benefit in terms of health^19^ and there is evidence of a decline in PA for children transitioning to adolescence^24^.

Compulsory schooling, and in particular, the school recess, are ideal contexts to promote PA since schools have integrated rest periods during school hours for the entire child and adolescent population^20^. Unless the administrator becomes aware of the need to improve physical education classes, in the current situation, these seem to have a limited potential to contribute to the recommended levels of PA^18^. Therefore, it is necessary to implement strategies that can help schools improve children’s sedentary tendency.

The interventions during recess can improve levels of PA^5,6^. Ridgers et al.^20^ reported that the recess period can contribute to 5% to 40% of the recommended levels for daily PA without any intervention. However, several strategies more or less structured have been implemented to increase the levels of PA for children during recess. They include, among others, providing an extra play equipment, painting play areas on the courtyard floor, putting up physical structures, providing facilities, adding a weekly activity for students, engaging teachers to promote activities or use active videogames^5,6,23^.

However, most of those strategies require additional budgets, which contrasts with the economic hardship of many educational centers and administrations. Unfortunately, the deficit of material is evident worldwide, even in the most developed countries and considering the current crisis^9^. Therefore, although it has been suggested that more strategies for boosting PA during recess^5^ must be implemented, it is necessary to align those strategies with economic opportunities.

In this sense, a viable strategy, although still unexplored, could be the self-construction of their own material to play. The approach based on self-constructed material attempts to involve the child in the manufacturing process of the resources they will use when playing^13,16,17^. Unlike paid material and equipment to play games, the self-constructed ones reuse or recycle worthless materials (among others, cardboard, newspapers or plastic bags) to transform them into toys at no or very little cost. Therefore, the promotion of the self-constructed material is of special importance on limited budgets and lack of equipment^13^. However, with the economic saving, other advantages should be emphasized, such as the increase in the participation time (in and out of school), the possibility of adapting the material to evolutionary development, the promotion of creativity and the cognitive implication for the children. A recent survey showed elevated levels of motivation and interest for the self-constructed materials among students of basic and pre-school education^14,15^.

Aware of this gap in research, this study aimed to analyze whether an intervention based on free play with self-constructed material increases the level of physical activity of students during recess.

## METHODS

### Participants

The study was conducted in a public school of a city in Northern Spain. The study population included 166 children in the third to the sixth grade of primary education. While data gathering, seven children were excluded from the analysis due to malfunction of the accelerometer and nine due to sick leave during the measurement. The parents of four children did not sign the authorization and so those children were not part of the study. We established as a criterion for inclusion at least three weekly records both pre-test and post-test. As a result, a sample of 146 children was evaluated, aged between nine and 12 years old (average = 10.64; standard deviation (SD) = 1.13). The intervention group was formed by 74 participants (36 boys and 38 girls, average age: 10.6 [SD = 1.14] years old) and the control group by 72 participants (38 boys and 34 girls, average age: 10.6 [SD = 1.13] years old). The school period had a recreational period of 30 minutes in the morning (from 11:00 a.m. to 11:30 a.m.) every day of the week. In these periods of recess, the children were not provided additional play material nor were any organized activities scheduled. Based on the basic principles of the Declaration of Helsinki, the study protocol was approved by the Ethical Committee of the Faculty of the corresponding University. Both the school administration and the parents or guardians of the students sent their informed consent.

### Research Project

We evaluated the levels of PA of the children through an accelerometer in a controlled project pre-test-post-test. The participants of each course were chosen at random in the intervention group and in the control group (chosen by conglomerates). During a session, the physical education teacher taught the intervention group students to build certain materials that were freely finished at home. In addition to the session, the teacher provided a document with instructions and guidelines to construct the material. Third and fourth-grade students of the treatment group built cardboard paddles (*paladós*) that were attached to the hands with rubber bands and newspaper balls stuck together, while the fifth and sixth-grade students built flying rings with cardboard and masking tape (like a frisbee). During the five days of one week, the intervention group children were allowed to play freely during recess with the material they each had built. A separate space was used to prevent contamination of the intervention with the control group. The teachers did not intervene, they only supervised the normal development of recess time.

### Instruments

The data concerning the date of birth and the gender of the participants were provided by the school.

For the anthropometry, the children were weighed without shoes and dressed before the pre-test and post-test with Roman scales, 100 g precision, ASIMED MB 201T (Asimed S.A, Barcelona, Spain). In the same way, they were measured before the pre-test and post-test with a rigid measuring tape, 0.1 cm precision, ASIMED MB 201T, direct reading (Asimed S.A, Barcelona, Spain).

To objectively measure the level of PA of the children, we used accelerometers ActiGraph-GT3X (ActiGraphTM, LLC, Fort Walton Beach, FL, USA). The data were collected through a triaxial function every 10 seconds. We excluded periods that had periods of 10 minutes of continuous zeros. The cut-off points were adjusted to the children’s age of the Freedson et al.^7^ to categorize the intensity of PA of the children as sedentary (SED) (0–149 counts per minute [cpm]), light (LiPA) (150–499 cpm), moderate (MPA) (500–3.999 cpm), and vigorous (VPA) (> 4,000). The result variables include the percentage of time devoted to the SED, LiPA, MPA, VPA, and moderate and vigorous (MVPA) activity during recess.

We measured the levels of PA of the children in both groups in the pre-test and post-test (three months later). Two fellows (a boy and a girl) of the research team, trained for this goal, put the accelerometers in the children every day in the morning (before the start of lessons) and collected them in the lunch recess. The accelerometer was put above the right hip and under the clothes by an elastic strap. To prevent children from increasing their activity level with the use of the accelerometer, they were only informed about the purpose of the measures after the post-test measurement. Pre-test and post-test measurements were organized in days with weather conditions that allowed outdoor play.

### Data Analysis

To download the files and analyze the data from the accelerometers, we used the software Actilife 6.7.1, the results of which were exported to the computer. We introduced the weight of each child in the pre-test and post-test measures. We selected the accelerometer data related to the recess and analyzed them with the SPSS program for Windows (19.0). To assess the effects of the self-constructed material on the levels of child PA during recess, we used a repeated measures ANOVA, with time (pre-test-post-test) as a determining factor and the group (intervention, control) as a factor between subjects. To search for the differences between boys and girls, the gender was included as a second factor between subjects (time × group × gender). The level of statistical significance was set at p < 0.05. We calculated the size of the effect^4^ (Cohen’s *d*).

## RESULTS

The descriptive data of the total sample in the pre-test showed that children participated in MPA at 35% (SD = 11) and in VPA at 10% (SD = 6) of the recess time (boys: MPA 38%, SD = 11, VPA 11%, SD = 6; girls: MPA 31%, SD = 9, VPA 9%, SD = 5). The Table shows the percentage of time invested in SED activity, LiPA, MPA, VPA, and MVPA during the school recess time for the intervention and control groups, in the pre-test and post-test measurements. Significant intervention effects were found in the five variables analyzed: SED (F = 38.19; p < 0.01), LiPA (F = 76.56; p < 0.01), MPA (F = 27.44; p < 0.01), VPA (F = 61.55; p < 0.01), and MVPA (F = 68.76; p < 0.01). In the intervention group, there was a significant decrease in the time invested in SED activity and LiPA in favor of a significant increment of the MPA, VPA, and MVPA compared to the control group. The effect size (d) was great in all variables, greater than 0.80 and especially high in the LiPA.

**Table:** Averages, standard deviations, F-values and effect size of the percentage of the time invested in distinct levels of physical activity during recess.

Variable		Pre-test			Post-test			F effect intervention	F differences gender	*d*
	
		Total sample Average % (SD)	Boys Average % (SD)	Girls Average % (SD)	Total sample Average % (SD)	Boys Average % (SD)	Girls Average % (SD)			
SED	Control	44.4 (14.93)	40.0 (13.04)	50.0 (15.05)	39.8 (16.86)	35.1 (16.89)	45.8 (15.07)	38.19^b^	1.78	1.34
	Intervention	40.8 (13.74)	36.1 (14.12)	45.4 (11.86)	22.2 (8.93)	20.2 (8.99)	24.1 (8.58)			
LiPA	Control	12.9 (2.99)	12.7 (3.05)	13.2 (2.96)	13.1 (2.71)	13.1 (2.42)	13.1 (3.09)	76.56^b^	3.23	2.50
	Intervention	12.7 (2.52)	12.3 (2.37)	13.1 (2.61)	8.3 (2.60)	7.1 (2.27)	9.4 (2.44)			
MPA	Control	33.7 (11.26)	37.1 (10.99)	29.5 (10.27)	37.0 (12.56)	40.1 (12.59)	33.0 (11.53)	27.44^b^	6.58^a^	1.22
	Intervention	35.7 (10.59)	39.8 (11.52)	31.8 (8.02)	48.8 (7.01)	47.7 (7.08)	49.8 (6.88)			
VPA	Control	9.0 (4.88)	10.3 (4.76)	7.4 (4.60)	10.1 (5.56)	11.7 (5.57)	8.1 (4.95)	61.55^b^	5.51^a^	1.25
	Intervention	10.8 (6.46)	11.9 (7.49)	9.7 (5.19)	20.7 (10.54)	24.9 (11.61)	16.7 (7.77)			
MVPA	Control	42.7 (13.94)	47.4 (12.58)	36.7 (13.55)	47.1 (16.65)	51.8 (16.86)	41.1 (14.56)	68.76^b^	0.85	1.65
	Intervention	46.4 (13.74)	51.7 (14.59)	41.5 (10.94)	69.5 (10.31)	72.7 (10.41)	66.5 (9.39)			

SED: sedentary activity; LiPA: light physical activity; MPA: moderate physical activity; VPA: vigorous physical activity; MVPA: moderate and vigorous physical activity; *d*: effect size (Cohen^18^)

^a^ p < 0.05

^b^ p < 0.01

Significant gender differences were shown (time x group x gender) for MPA (F = 6.58; p < 0.05) and VPA (F = 5.51; p < 0.05). The decrease between the pre-test and post-test in the experimental group in SED activity or LiPA was similar in boys and girls. However, although the increments of MPA and VPA were significant, both in boys and in girls of the experimental group between the phases before and after, the girls showed an increase greater than the boys in MPA, and the boys had a significantly greater increase in VPA compared to the girls.

## DISCUSSION

The objective of this research was to evaluate the effects of playing with the self-constructed material in the levels of PA of children during recess. In the pre-test, the children became involved in the MVPA slightly less than half of the recess time (42%–46%). These values were very similar to those reported in previous studies^22^ in different geographical areas.

The results of this study showed that the self-constructed material was effective to increase levels of PA of children during recess. On the one hand, decreasing SED activity and LiPA, and on the other, increasing the time devoted to MPA, VPA, and MVPA in the total sample. In the absence of prior studies with the self-constructed material, the results are compared with studies that have implemented interventions with marketed or recycled material. Our results converge with previous studies^1,8,12,21,25,26^ that concluded that providing equipment for additional playing during recess is effective to increase the levels of PA for the children. However, Cardon et al.^2^ found no PA increments when providing additional play equipment to preschool students. It is possible that preschool students need a different type of equipment or greater supervision by the teaching staff due to their lack of autonomy to play actively^6^. In addition, the study by Verstraete et al.^25^ concluded that the play equipment provided for recreational time were effective in increasing the levels of MPA and MVPA, but not VPA. In this research, the self-constructed materials caused increments, even in VPA, certainly because they were challenging and suggestive for this sample of students. In fact, the levels of MVPA (close to 70% of the recreational time) in this study with the self-constructed material are higher than those described in other studies that have examined the conventional material supply strategy. Verstraete et al.^25^ reported values above 53% in MVPA in students from the fifth to sixth grade of elementary school. Other studies in primary education have found that, except for balls, the availability of equipment was not a significant predictor of PA^28^, or even a reduction in MVPA of children with a healthy weight (< 85 percentile)^10^.

A possible explanation for the results is the quantity and characteristics of the material, which satisfies the students’ interests. The fact that each participant could have their own material without turns, rotations, or waiting, that the equipment was adjusted for the development, and even that it had been transformed and “customized” for their own interests (for example, choosing the shape, color, images in the paddles), might have increased the motivation of the children and encouraged more PA. The choice of the type of material to be built can be a key element for the successful promotion of PA. Thus, the paddles and the rings were challenging and promoted PA levels for children of both sexes.

On the other hand, the results of this study confirmed previous findings that boys are more active than girls during non-structured recess^25^. Interestingly, the girls in this study had a greater increase in their level of MPA, whereas boys showed a greater increase in VPA. Prior research has shown that boys tend to be more competitive and be more focused on winning competitive events than girls^3^, which can cause them to get involved with greater intensity in the game. Regardless, the results of this study related to girls give us a lot of hope, since they are positive and healthy for the traditionally more sedentary gender^3^.

In conclusion, compared with no intervention, the strategy of using self-constructed material during recess has the potential to promote short-term MVPA in boys and girls. However, a limitation of the study is that the data were obtained in a single primary school and for one week. Longitudinal studies are needed to monitor the effect of fatigue described in other studies when using the same material for a while. In this case, we suggest a study of what kind of equipment should be built and for how long they should be used to maintain high levels of PA and the children’s motivation. Moreover, the effect of similar projects during the lunch recess and in an extracurricular context should be analyzed.
